# Juvenile hormone controls ovarian development in female *Anopheles albimanus* mosquitoes

**DOI:** 10.1038/s41598-019-38631-6

**Published:** 2019-02-14

**Authors:** Salvador Hernández-Martínez, Víctor Cardoso-Jaime, Marcela Nouzova, Veronika Michalkova, Cesar E. Ramirez, Francisco Fernandez-Lima, Fernando G. Noriega

**Affiliations:** 1 Centro de Investigaciones Sobre Enfermedades Infecciosas, Instituto Nacional de Salud Pública, Cuernavaca, Morelos Mexico; 2 Department of Biological Sciences and Biomolecular Science Institute, Florida International University, Miami, FL USA; 3 Institute of Parasitology, Biology Centre CAS, Ceske, Budejovice Czech Republic; 4 Department of Chemistry and Biochemistry, Florida International University, Miami, USA

## Abstract

Anophelinae mosquitoes are vectors of human malaria, a disease that infects hundreds of millions of people and causes almost 600,000 fatalities annually. Despite their medical importance, laboratory studies on key aspects of Anophelinae reproductive biology have been limited, and in particular, relatively little is known about the role of juvenile hormone (JH) in the control of female reproduction. The study presented here attempts to fill a gap of knowledge in our understanding of the JH control of ovarian development in female Anophelinae mosquitoes, using *Anopheles albimanus* as a model. Our studies revealed that JH controls the tempo of maturation of primary follicles in *An. albimanus* in a similar manner to that previously described in *Aedes aegypti*. At adult eclosion JH hemolymph titer was low, increased in 1-day old sugar-fed insects, and decreased in blood fed individuals. JH titers decreased if *An. albimanus* females were starved, and were reduced if insects emerged with low teneral reserves, precluding previtellogenic ovarian development. However, absolute hemolymph titers were lower than *Ae. aegypti*. Decapitation experiments suggested that if teneral reserves are sufficient, factors from the head activate JH synthesis by the *corpora allata* (CA) during the first 9–12 h after adult emergence. In conclusion, our studies support the hypothesis that JH controls previtellogenic ovarian development in female *An. albimanus* mosquitoes, in a similar manner that have been described in Culicinae.

## Introduction

Despite their importance as vectors of malaria and filariasis, laboratory studies on key aspects of Anophelinae biology have been limited, and in particular, relatively little is known about the role of juvenile hormone (JH) in the control of female reproduction. In contrast, the hormonal control of female reproductive biology has been extensively studied in *Aedes aegypti*^1^. Therefore, many assumptions have been made on *Anopheles* species by extrapolating studies from *Ae. aegypti*. Physiological and behavioral findings cannot always be extrapolated from Culicinae to Anophelinae, because the underlying physiological mechanisms are quite different^2,3^. The study presented here attempts to fill a gap of knowledge in our understanding of the JH control of nutritional allocation and ovarian development in female Anophelinae mosquitoes, using *Anopheles albimanus* as a model.

Mosquitoes have polytrophic meroistic ovaries. The anterior part of each ovariole contains follicle stem cells and germline cells (germarium), and the rest of the ovariole (vitellarium) contains differentiated follicles (ultimate or primary and penultimate or secondary), each originally comprising an oocyte and seven nurse cells surrounded by a follicular epithelium^4^. There are three major periods in the ovary development during a gonotrophic cycle in mosquitoes: previtellogenesis (PVG), ovarian resting stage (ORS) and vitellogenesis (VG)^4^. In *Ae. aegypti*, JH III, synthesized by the *corpora allata*, directly controls nutrient allocation into the ovaries in the PVG and ORS phases^5^, and indirectly influences the fate of VG follicles after a blood meal^6^.

*Anopheles albimanus* is the main malaria vector species in Central America, northern portions of South America and the Caribbean. The distribution of *An. albimanus* ranges from Mexico to northern Peru on the Pacific coast, and from Texas to Venezuela on the Atlantic coast. This species can also be found in South Florida and on most of the Caribbean islands^7^.

Pioneering works described changes in ovary and follicle developments before and after a blood meal and/or after oviposition in several species of *Anopheles*^8–10^; including some aspects of egg development in *An. albimanus*, which presents between 50–95 developing follicles per ovary^11^. Egg development in *An. albimanus* and *Ae. aegypti* shows similarities; including the growth of the primary and secondary follicles before and after a blood meal, as well as the presence of a 20-OH-ecdysone (20E) peak in close correlation with a peak of vitelline production. This early work on *An. albimanus* focused primarily on post-blood meal development, and did not explore in detail previtellogenic development or the role of JH mediating reproductive development. In this study, we investigated the role of JH in the control of previtellogenic ovarian development in female *An. albimanus*. Our results revealed that JH controls the tempo of maturation of primary follicles in *An. albimanus* in a similar manner to that described in *Ae. aegypti*. JH titers decrease if *An. albimanus* females are starved, and are also reduced if insects emerge with low teneral reserves, precluding previtellogenic ovarian development. However, absolute hemolymph titers are lower than *Ae. aegypti*. Decapitation experiments suggested that if teneral reserves are sufficient, factors from the head activate JH synthesis by the *corpora allata* (CA) during the first 9–12 h after adult emergence.

## Results

### Follicle development in sugar-fed large and small females

Wing length is a reliable indicator of mosquito size and nutritional status^12^. The average wing length from our large laboratory-reared *An. albimanus* females was 3.242 ± 0.006 mm (n = 110). Through nutritional limitation of larvae, we generated small laboratory-reared mosquitoes with a wing length average of 2.828 ± 0.020 mm (n = 20 females) (see Supplemental Fig. S1). We compared our laboratory-reared small and large females with female *An*. *albimanus* collected in two close locations in the state of Chiapas (Mexico). Pico de Oro and Frontera Corozal are very similar habitats separated by a distance of 35 miles. Field-collected females from Pico de Oro captured during the rainy season (October 2017) had wing lengths of 3.480 ± 0.059 mm (mean ± SEM, n = 10), while females collected at Frontera Corozal during the dry season (February 2018) had wing lengths of 2.496 ± 0.084 mm (mean ± SEM, n = 10) (see Supplemental Fig. S1).

**Figure 1:**
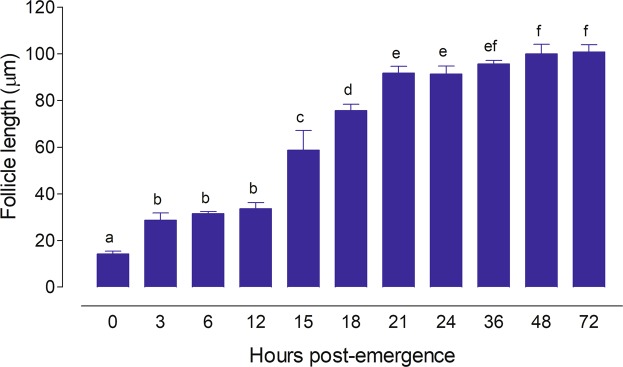
Ovarian development in 10% sugar-fed *An. albimanus* females. The lengths of the terminal follicle were measured from newly emerged females until 3 days after emergence. Each bar represents the mean ± SEM of follicle lengths from ten females, 10/follicles each (at least 100 independent determinations). Values labeled with different letters are significantly different by Tukey’s test after ANOVA at *P* < 0.001.

**Figure 2:**
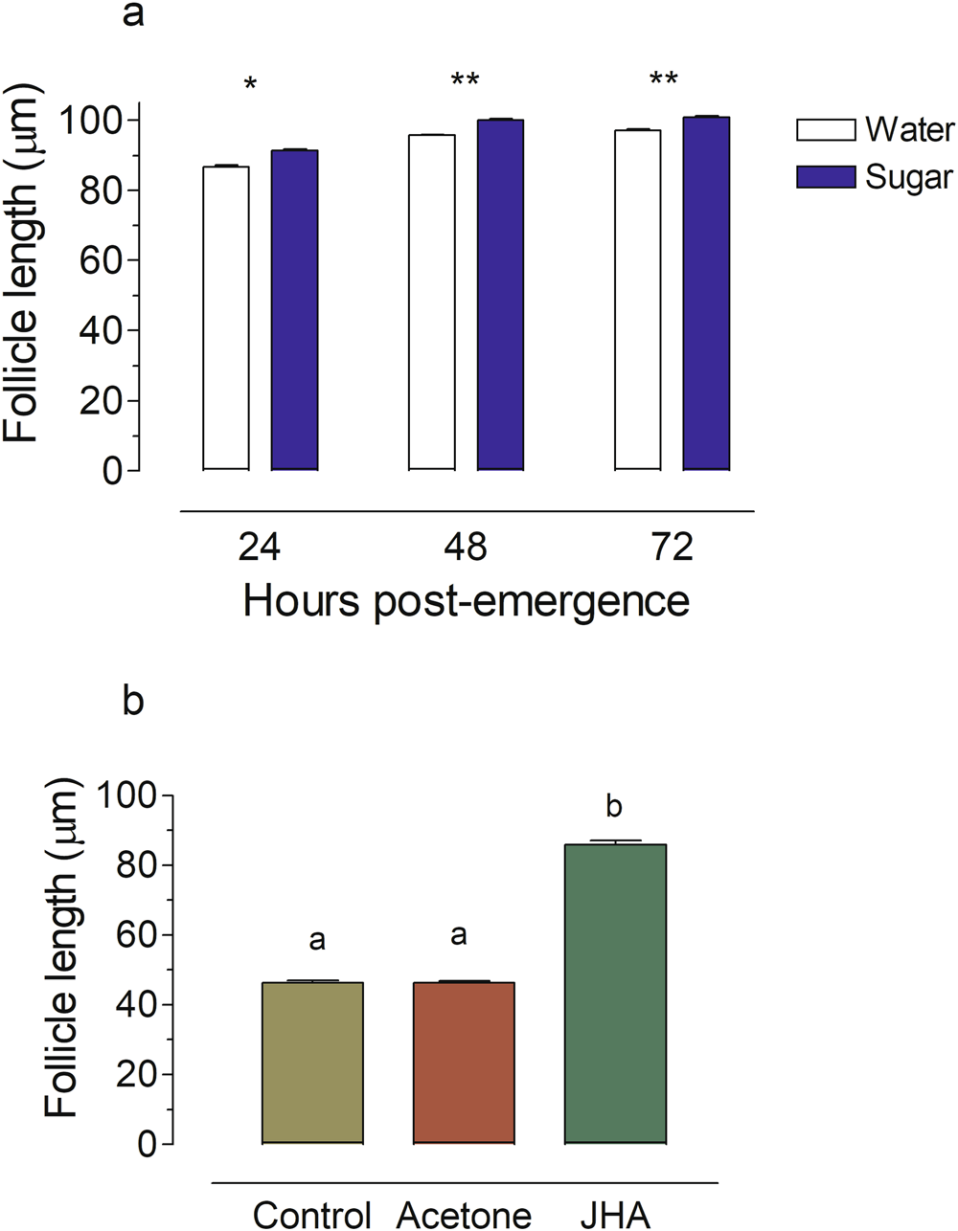
Ovarian development in water-fed and small *An. albimanus* females. (**a**) Effect of sugar-deprivation. The lengths of the terminal follicles were measured at 1, 2 and 3 days after emergence in water- and sugar-fed females. Each bar represents the mean ± SEM of follicle length from ten females, 10/follicles each (at least 100 independent determinations). Asterisk denotes significant difference (unpaired t-test; **P ≤ 0.01, *P ≤ 0.05). (**b**) The lengths of the terminal follicles were measured at 24 after emergence in small females emerged with low teneral reserves (control). Acetone or Methoprene (JHA, 1 ng/µl) were topically applied 12 h after emergence, and follicles were examined at 36 h. Values labeled with different letters are significantly different by Tukey’s test after ANOVA at *P* < 0.001.

Large adult laboratory-reared females emerged with 93.8 ± 3.4 follicles (n = 10 females) per ovary, while small females have roughly a half of this number (45.6 ± 2.6 follicles (n = 5 females) (P < 0.0001). The average primary follicle length from laboratory-reared newly emerged (0 h) large and small females was ~15 µm. In large females fed a 10% sugar meal since adult emergence, primary follicles grew to up to ~40 µm by 3 h post-emergence, and remained the same size until 12 h (Fig. 1). After 12 h, these follicles started to grow to reach a final length of up to ~100 µm by 24–36 h (“resting stage size”) (Fig. 1). The primary follicle lengths of large water-fed females was slightly smaller, but also reached the “resting-stage” length (~100 µm) by 24–36 h after adult emergence (Fig. 2a). All females died if kept for 4 days with only water.

The primary follicles of sugar-fed small mosquitoes had an average length of ~45 µm at 36 h after emergence (Fig. 2b), and consequently did not reach the resting-stage size, unless Methoprene, a JH analogue (JHA), was topically applied.

### Ovarian development in large blood-fed females

**Figure 3:**
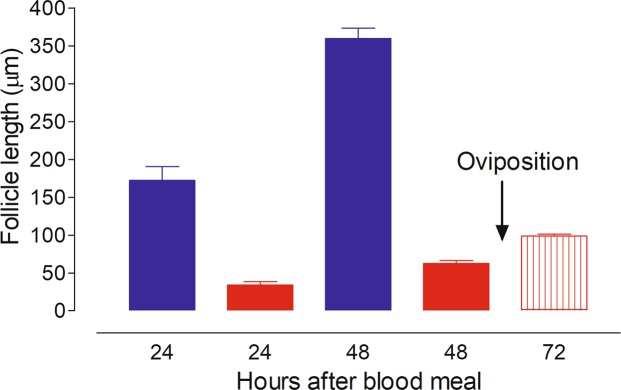
Ovarian development in blood-fed *An. albimanus* females. The follicle lengths in the 1^st^ and 2^nd^ gonotrophic cycle were measured at different times after blood-feeding. Oviposition (arrow) indicates the point in which eggs were laid. Primary follicles in the first gonotrophic cycle (blue). Secondary follicles in the first gonotrophic cycle (solid red). Primary follicle in the second gonotrophic cycle (striped red). Each bar represents the mean ± SEM of follicle length from ten females, 10/follicles each (at least 100 independent determinations).

Changes in lengths of the primary and secondary follicles were measured at different times after providing a single blood meal. By 24 h after blood feeding, the primary follicle from the first gonotrophic cycle has reached a size of ~180 µm, while the secondary follicle measured ~35 µm (Fig. 3). At 48 h after blood feeding, the primary follicle grew to ~360 µm, and the secondary follicle grew to ~62 µm. By 72 h, the primary follicles developed into chorionated eggs that were laid, while the former “secondary follicles” had matured into fully developed resting-staged primary follicles of the second gonotrophic cycle (Fig. 3).

### Ovarian development in decapitated large mosquitoes

**Figure 4:**
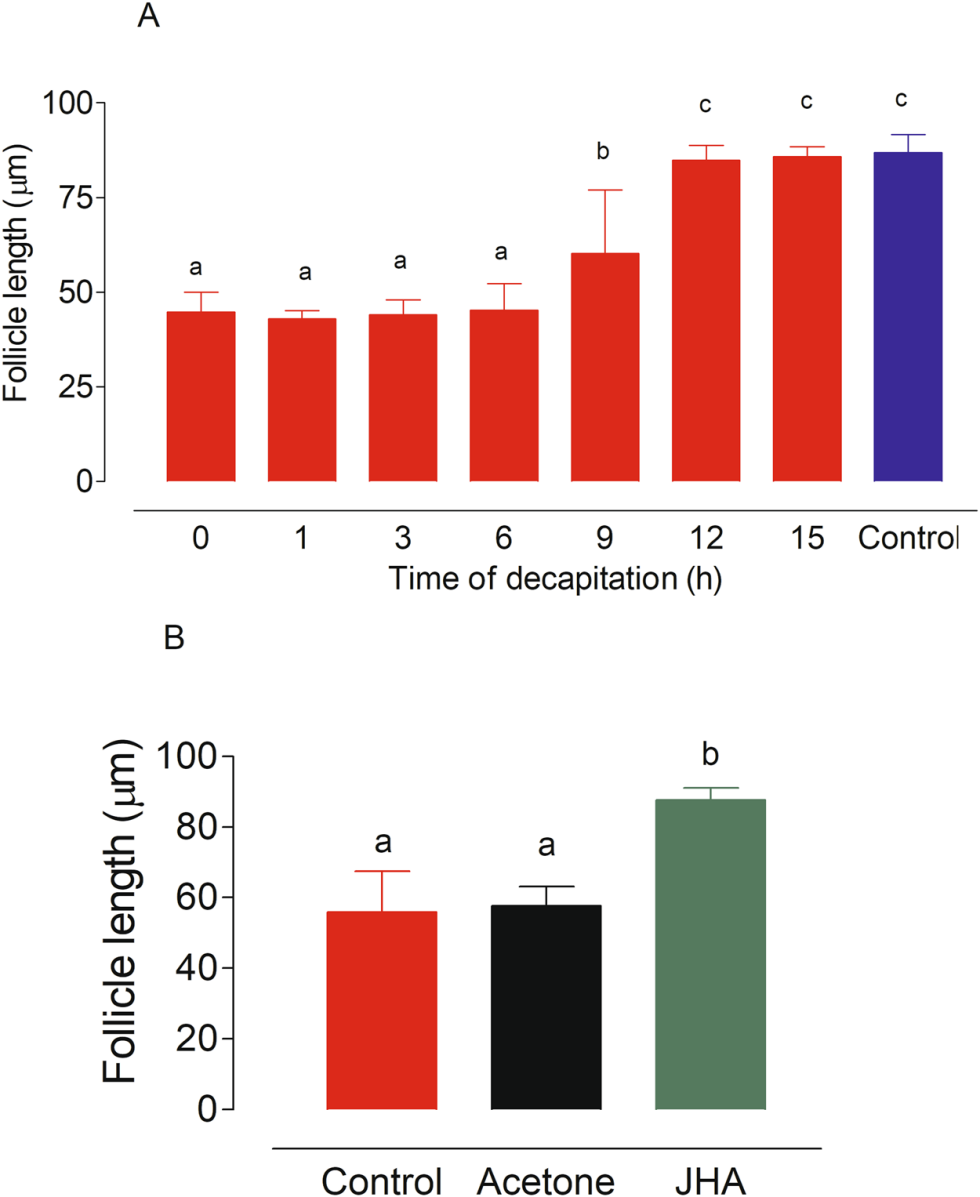
Ovarian development in large *An. albimanus* females decapitated at different times after adult emergence. Mosquitoes were decapitated and kept in a humid chamber. Follicle development was evaluated at 48 h after emergence. (**A**) Effect of time of decapitation on follicle development. Controls were not decapitated. (**B**) Effect of Methoprene on follicle development in decapitated females. Females were decapitated at adult emergence, and Methoprene, a JH analogue (JHA) or acetone, were topically applied 12 h later. Controls were just decapitated. Each bar represents the mean ± SEM of follicle length from ten females, 10/follicles each (at least 100 independent determinations). Values labeled with different letters are significantly different by Tukey’s test after ANOVA at *P* < 0.001.

Mosquitoes were decapitated at 0, 1, 3, 6, 9, 12 and 15 h after adult emergence. At 48 h after emergence, ovaries were dissected, and follicle development was evaluated. Decapitations of females during the first 6–9 h after emergence prevented the completion of the previtellogenic development of the follicles (Fig. 4A). Factors from the head were necessary only during the first 12 h post-emergence, after this period, decapitation did not prevent previtellogenic follicles to reach the “resting stage size” (~100 µm) (Fig. 4A).

Another group of females was decapitated at adult emergence (0 h), and Methoprene was topically applied 12 h later. Mosquitoes were kept in a humid chamber and follicle length was evaluated at 48 h post emergence (Fig. 4B). Methoprene induced a significant increase in the length of previtellogenic follicles in decapitated females when compared with those observed in control or acetone-treated females (Fig. 4B).

### Survivorship of decapitated large females

**Figure 5:**
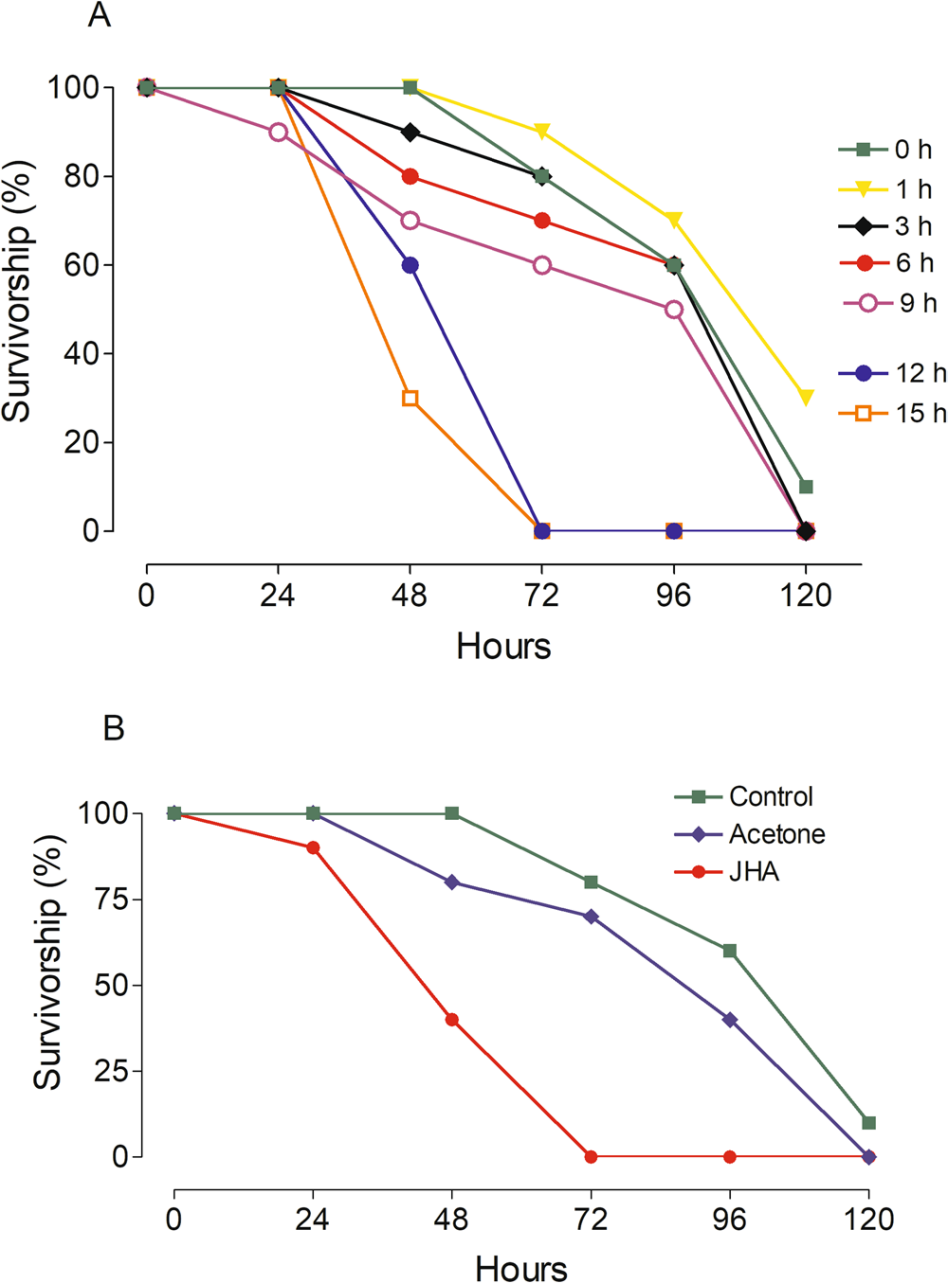
Survivorship of decapitated females. (**A**) Groups of 20 females were decapitated at 0, 1, 3, 6, 9, 12 and 15 h after emergence, and survivorship was evaluated every day. (**B**) Groups of 20 females were decapitated at adult emergence (0 h) and after 12 h were treated with either acetone or Methoprene, and survivorship was evaluated every day. Controls were just decapitated. Each data point represents the percentage of insects alive.

Groups of females were decapitated at different times after emergence and survivorship was evaluated every day. Decapitated females were unable to feed or drink, and therefore had to survive on the nutritional resources stored at the time of decapitation. There was a significant decrease in survivorship when females were decapitated at 12 h or later post-emergence, a time when head factors have been already released to direct the allocation of nutritional resources towards previtellogenic ovarian development (Fig. 5A). In a complementary experiment, groups of females were decapitated at adult emergence (0 h), and after 12 h were treated with either acetone or Methoprene. Topical application of the JHA significantly decreased the survivorship of decapitated females (Fig. 5B).

### JH III hemolymph titers in large and small females

**Figure 6:**
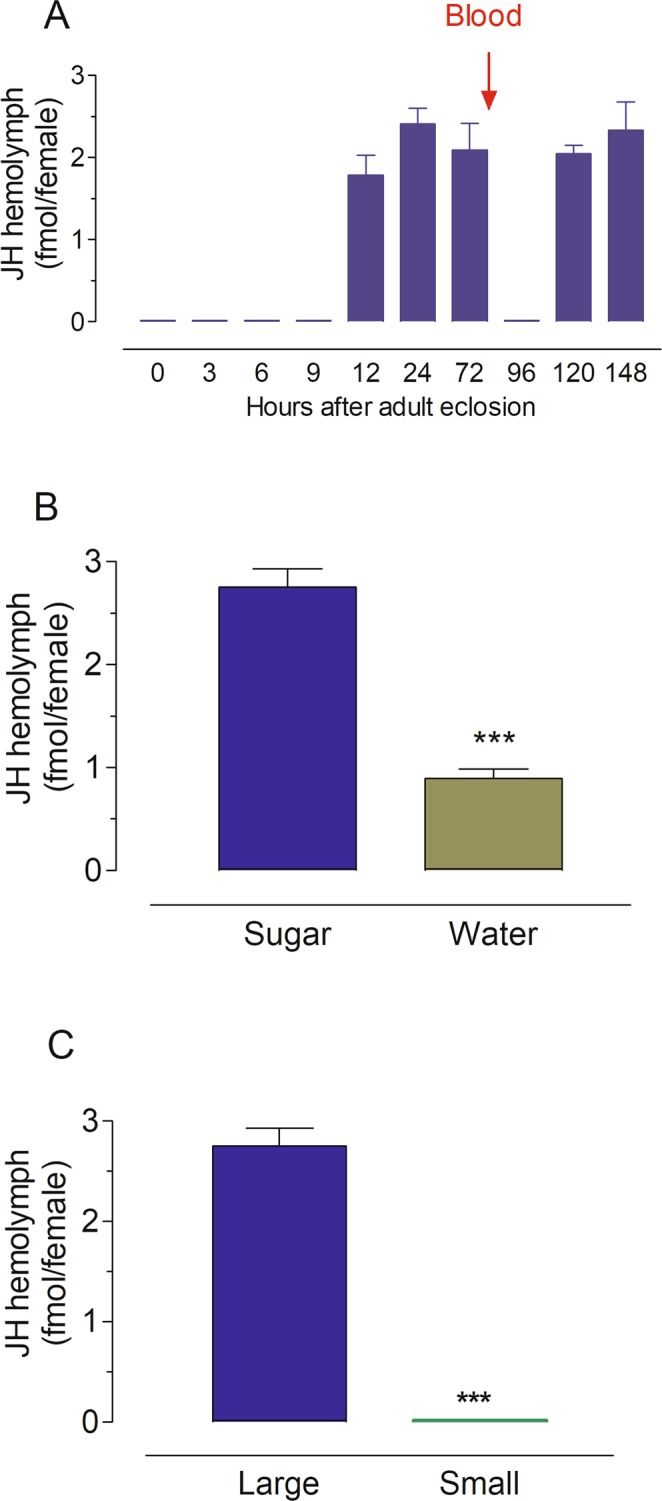
JH hemolymph titers in *An. albimanus* females. (**A**) JH titers in sugar-fed females and after blood-feeding. Mosquito hemolymph was obtained from sugar-fed females at different times after adult emergence, as well as at 24 and 48 h after blood-feeding (BF). (**B**) JH titers in the hemolymph of water-fed and sugar-fed females at 24 h after emergence. (**C**) JH titers in the hemolymph of large and small females at 24 h after emergence. JH titers are expressed as fmol/female. Each data point represents the mean ± SEM of at least four independent replicates of groups of 5 females.

Hemolymph was obtained by perfusion^13^, and JH III titers were measured using a high performance liquid chromatography coupled to electrospray tandem mass spectrometry approach (HPLC-MS/MS)^14^. The changes in the titers of JH detected in hemolymph of sugar-fed females after adult emergence, as well as following a blood meal are presented in Fig. 6A. Titers were undetectable in newly emerged females and only significantly increased by 12 h after adult emergence. They remained relatively constant with titers around 2-3 fmol/female during the first three days in sugar-fed large females. JH titers decreased after blood feeding, and were almost undetectable 24 h after a blood meal. Titers increased again by 48 h after blood meal, reaching values similar to those detected before a blood-meal. If after emergence large females were given only water, JH titers 24 h later were significantly reduced when compared with sugar-fed females (Fig. 6B). JH titers 24 h after emergence were compared in small and large mosquitoes. JH titers were significantly reduced (below detection levels) in females raised under nutritional limitation and emerging with low teneral reserves (small females) (Fig. 6C).

## Discussion

The correct allocation of nutrients between competing needs such as maturation, growth, reproduction or dispersal by flight is a vital component of a mosquito life-history strategy^1^. Nutritional homeostasis in a female adult mosquito depends on its ability to acquire, store and manage nutrients. Adult female reserves originate from larval accrual (teneral reserves), as well as from sugar- and blood-meals taken by the adult. The ability to manage and store nutrients is related to the biology and ecology of each species, and affects reproductive development and reproductive capacity.

Oogenesis in mosquitoes is a nutrient-limited process, initiated only if sufficient nourishment is available^15^. After eclosion, female mosquitoes enter a “maturation period” that lasts one to two days. This very important period encompasses anatomical differentiation, behavioral changes, activation of hormonal systems, maturation of the digestive and reproductive systems, as well as development of the flight muscles. Successfully carrying out all these processes is critical before an anautogenic female mosquito (those that need a blood meal to develop eggs) becomes competent to blood feed and lay eggs. For the completion of this maturation period, the quantity of teneral reserves “inherited” from the larval stages is crucial^15^.

Culicinae mostly utilize containers or very nutrient rich standing and ephemeral water; habitats that almost no other organism can utilize effectively; this facilitates the gathering of nutrients, necessary to have large teneral reserves, and promotes gonotrophic concordance with blood meals (one blood-meal leads to the maturation of a single batch of eggs). On the other hand, Anophelinae mosquitoes are more likely to be found in permanent bodies of water like swamps and lakes; habitats less rich in nutrients and occupied by many more competitors. In addition, Anophelinae larvae are often incapable of accumulating abundant teneral reserves, even when food is plentiful. They lack a siphon, and for that reason spend most of their lives at the water surface^16^. Harvesting primarily the water surface for floating food particles is less rewarding nutritionally than browsing on bottom detritus or suspension feeding^16,17^. Consequently, these constraints result in lower teneral reserves, and strongly affect the life and reproductive capacity of the adult Anophelinae female^2^.

Adult female *Anopheles* appear to emerge “undernourished” when compared with *Ae. aegypti*^2,3^. Consequently, Anophelinae females must feed on sucrose or blood rather frequently to be able to increase the amount of lipids stored. Females often feed on a host within the first 6–10 h of imaginal life^2^. Multiple blood meals within a single gonotrophic cycle (gonotrophic discordance)^18^ are common in Anophelinae mosquitoes^19,20^.

Reserve homeostasis is lower in *Anopheles*^2,15^, and females seem to be in constant need for subsequent blood meals to improve fecundity or to increase reserves. In contrast, *Ae. aegypti* often emerges with most of the nutrients necessary to develop a first batch of eggs and mostly requires a blood meal to “refill” the reserves expended, thus requiring tight and hormonally regulated concordance between blood meals and egg development^21,22^.

Another important aspect of the *Ae. aegypti* gonotrophic concordance is the strict control of the maturation of the secondary follicle, which only differentiates and separates from the germarium stimulated by a rise of 20E titer after a blood meal. Similarly, the injection of 20E into unfed *Ae. aegypti* females caused secondary follicles to separate from their germariums in many ovarioles^23^. On the contrary, in *An. albimanus*, we often observed the separation and development of secondary follicles to the ~40 µm size in sugar-fed large females by 24–36 h after emergence, when the primary follicles have only reached their maximum “resting stage” size of 100 µm (see Supplemental Fig. S2). Briegel and Hörler^20^ also observed ovarioles of *An. albimanus* with secondary maturing oocytes on top of the primary mature ones.

Hormones from the CA, neurosecretory system and the ovary regulate the major steps in oocyte maturation in mosquitoes^24^. In *Ae. aegypti*, the alternation of high titers of JH with low 20E before blood feeding, and low titers of JH with high 20E after a blood meal, assures the development of a single follicle (the primary) per ovariole in each gonotrophic cycle, preventing the simultaneous development of primary and secondary follicles.

Juvenile hormone is a key mediator in trade-offs between survival and reproduction in mosquitoes^5,6^. There is a positive relationship between teneral reserves derived during larval stages and CA activity post-emergence; the biosynthetic activity of the CA is drastically reduced in *Ae. aegypti* females emerged with low teneral reserves^25^. Decapitations, *corpora allata* (CA) removal and abdominal ligations have also been used to prove that the growth of the previtellogenic follicles is under control of factors from the brain and CA in anautogeneous mosquitoes^26–29^.

In the present study, we analyzed in detail the previtellogenic follicle development in *An. albimanus*. A striking difference with *Ae. aegypti* is the small size of the primary follicle in newly emerged females (15 µm vs 40 µm in *Ae. aegypti*), as well as the substantial growth that occurs during the first 3 h of adult life in *An. albimanus*. This “initial” growth or “first-phase” is independent of factors from the head, as it cannot be prevented by decapitation. Most likely, this is JH-independent process, that is somehow equivalent to the growth or “differentiation” of the primary follicle in the pharate adult or late pupae of *Ae. aegypti*; this process that involves the formation of new follicles within the germarium, as well as their separation from it, is regulated by 20E in *Ae. aegypti*^23^.

Completing the follicle previtellogenic development is a nutrient-dependent process in mosquitoes. Both, water-fed and sugar-fed *An*. *atroparvus*, reached the resting stage size with mean follicle lengths of ~90 μm^30^. Similarly, large *Ae. aegypti* activated the CA, and completed the previtellogenic development of follicles when raised on water only^25^. Briegel^2^ described that when *An. albimanus* of different body sizes were blood fed to repletion, only 12% of the females with wing lengths <2.95 mm developed eggs. On the contrary, 91% of those with wing lengths >3.05 mm were oogenic. A body size threshold corresponding to wing lengths of ~3 mm in *An. albimanus* and *An. gambiae* appears to be crucial for completing oogenesis with the first blood meal^2^. Large *An. albimanus* females, with wing lengths over 3.2 mm, carried considerable teneral reserves^15^, while *An. albimanus* females with wing lengths under 3.0 mm will use the first blood meal for the synthesis of maternal lipid and protein reserves, instead of making yolk, hence compensating for their low amount of teneral reserves^2^. In our experiments, the average wing length size of large females was 3.24 mm. Therefore, it was not surprising that these females were able to complete the second phase of previtellogenic development, and reach a maximum resting stage follicle size even when they were raised just with water. In contrast, as previously described in *Ae. aegypti*^25,29^, the primary follicles of sugar-fed small *An. albimanus* had an average length of ~45 µm at 36 h after emergence, and consequently did not reach the resting-stage size, unless Methoprene was topically applied.

In *Ae. aegypti* females the CA is inactive for most of the duration of the pupal stage^31,32^. As the anti-metamorphic role of JH ends, the CA of the late pupa (or pharate adult) is reactivated and begins to synthesize JH, which would now play an essential role orchestrating reproductive maturation^1,24^. During a gonotrophic cycle, JH hemolymph levels fluctuated sharply between *Ae. aegypti* females of different physiological conditions. At adult eclosion JH hemolymph titer was low, increased in 1-day old sugar-fed insects, and decreased in blood fed individuals^13^. Our studies are revealing a similar general pattern of JH changes in the hemolymph of *An. albimanus*, with undetectable levels in newly emerged females, higher JH titer in sugar-fed and very low JH after blood-feeding. These results suggest that the role of JH controlling the tempo of maturation of primary follicles in *An. albimanus* is similar to that described in *Ae. aegypti*.

The absolute values of JH detected in the hemolymph of mosquitoes are influence by many factors, including the strain, insectary rearing conditions, and quantification protocol used. When quantified by HPLC-MS/MS, titers, expressed as fmol/female, were significantly lower in *An. albimanus* when compared with *Ae. aegypti* (about 15fmol/female at 24 h). The amount of JH present in a µl of hemolymph, also depends on species-specific factors such as body sizes and hemolymph volumes. Hemolymph volumes in *An. stephensi* females fluctuated between 0.2 and 0.34 µl^33^, while in *Ae. aegypti* females varied between 1.0 and 2.0 µl^34^; so the final effective concentration of JH at the target cell (in fmol/µl) might be similar in the hemolymph of Culicinae and Anophelinae species.

Mosquito CA activity is controlled by factors present in the head^35^, and nutritional signals affect their release resulting in the activation or inhibition of JH synthesis. In *Ae. aegypti*, decapitation within 1–6 h after emergence or CA removal soon after eclosion prevents ovarian previtellogenic growth. Topical application of a JH analog stimulates normal growth of previtellogenic ovaries in decapitated or CA-ablated teneral females^27–29^. We confirmed the roles of the brain and JH on *An. albimanus* ovarian development by topically applying a JHA, which stimulated previtellogenic ovarian growth in decapitated mosquitoes. Follicles of these Methoprene-treated decapitated females attained normal previtellogenic growth. Our decapitation experiments suggested that the nutritional activation of the CA in *An. albimanus* also occurs during the first 9–12 h after emergence.

The correct allocation of nutrients between survival and reproduction is critical for female mosquitoes. Under starvation conditions with only access to water, all *An. gambiae* died within 4 days^30^. We observed a similar response in our studies. Decapitated large females that do not develop their ovaries are able to relocate limited teneral resources and survived longer.

An important question is how relevant are these laboratory studies when comparing with field populations of *An. albimanus*. While mosquito reared in the laboratory can be provided an optimal diet; in the field, mosquitoes often encounter suboptimal conditions that result in a high variability in teneral size and reproductive potential^12^. We compared *An. albimanus* collected in two close locations in the state of Chiapas (Mexico). Pico de Oro and Frontera Corozal are very similar habitats separated by a distance of 35 miles. Females collected at Pico de Oro during the rainy season were larger than females collected at Frontera Corozal during the dry season. The differences between these field-collected large and small mosquitoes are even bigger than those we generated in the laboratory, and most likely nutritional allocation and ovarian development in females from the two populations mirrored those described for the large and small females in our laboratory studies. Based in our results, we would predict major differences in teneral reserves, JH titers and oogenesis between these 2 mosquito populations. Mosquitoes in the rainy season have larger teneral reserves and reproductive capacity, with potentially major effects on malaria transmission. It has been described in Mexico^36^ and Africa^37^ that the onset of the rains brings a rapid explosion in mosquito numbers and a concomitant increase in malaria.

In conclusion, our studies support the hypothesis that JH controls nutritional allocation and ovarian development in female *An. albimanus* mosquitoes, in a similar manner that have been described in Culicinae^1^. However, biological and ecological differences often limit the ability of Anopheline larvae to build large teneral reserves, conditioning the reproductive biology of adult females. Female *An. albimanus* have reproductive strategies that seems to be a “transition” between continuous feeder dipterans like *Drosophila melanogaster*, where oogenesis is asynchronous, each ovariole contains many developing oocyte at different stages and eggs are produced continuously^38^, and discrete feeding dipterans such as *Ae. aegytpi* with paused and highly synchronous egg development.

In *Ae. aegytpi*, follicle resorption plays a key role adjusting reproductive output to nutrition^39^. Females develop simultaneously a complete batch of primary follicles, and are reluctant to “invest” nutrients in secondary follicles until the 1^st^ blood meal is digested. Accordingly, after blood feeding, it resorbs numerous primary follicles to match the available nutrition^6^. In Anophelinae, egg development might be less dependent on reserves and more closely tied to incoming nutrition provided by both sugar and blood meals; if nutrients are scarce females might resorb a few primary follicles, and pause secondary follicles development^20^.

## Methods

### Insects

*Anopheles albimanus* of the Tapachula strain, were raised at 28 °C, 80% relative humidity, with a 12-h light/12-h dark photoperiod at the National Institute of Public Health in Cuernavaca, Morelos, Mexico. Large or small mosquitoes were obtained by changing the number of larvae and amount of diet added to the pan.

#### Large mosquitoes

Larvae were reared in round pans (35 cm diameter X 15 cm high) containing 4 liters of water (200 larvae in an area of 960 cm^2^). Larvae were fed daily with either 100 (day 1-2), 200 (day 3-4) or 400 mg (5–7 day) of finely grinded kitten cat food (Whiskas). Most pupae appeared at day 8. Most females emerged by day 10, and only those with wing length of 3.15 mm or more were used.

#### Small mosquitoes

Larvae were reared in the same conditions as large insects, but the density was increased to 480 larvae/pan. Larvae were fed every other day with either 100 (day 1), 200 (day 3) or 400 mg (day 5 and 7-8) of food. Under such conditions, most pupae appeared at day 10. Most females emerged by day 12, and only those with wing length under 2.9 mm were used.

Adult females were fed *ad libitum* with cotton pads soaked on a 10% sucrose solution. Three-day-old female mosquitoes were fed once using an artificial-feeder and pig blood equilibrated to 37 °C. Adenosine-tri-phosphate (ATP) was added to the blood meal to a final concentration of 1 mM immediately before.

### Analysis of follicle length

Mosquitoes were isolated at adult emergence and kept under the conditions described above. Females were immobilized by brief exposure to ice and ovaries were isolated by tearing the intersegmentary membrane between the seventh and eighth abdominal segments, and placing the terminal segment in a drop of phosphate buffer saline (PBS, 19 mM NaH_2_PO_4_, 8.1 mM Na_2_HPO_4_, 154 mM NaCl, pH 7.0). Follicle length was measured under a dissecting microscope using an ocular micrometer.

### Analysis of survivorship and follicle length on decapitated mosquitoes

Mosquitoes were immobilized by brief exposure to ice and decapitated with a blade at different times after adult emergence (0, 1, 3, 6, 9, 12 and 15 h). Decapitated mosquitoes were kept at room temperature in a humid chamber. Ovaries were isolated as described above and follicle lengths measured using an ocular micrometer. Survivorship was recorded every day for a total of five days. Methoprene (1 ng/µl) was topically applied (0.5 µl) on mosquito abdomens.

### Hemolymph collection

Mosquito hemolymph was obtained by perfusion as previously described^13^. Briefly, the abdomen of chilled mosquito was washed with 70% ethanol and air-dried. Fine needles (made from 100 μl micro-glass capillary tubes using a pipette puller P-30, Sutter Instrument, Novato, CA) mounted in a pipette pump (Drummond, Broomall, PA) were inserted manually through the neck membrane into the thoracic cavity. Insects were perfused with 20 µl of a “bleeding solution” of phosphate-buffered saline (PBS) (100 mM NaCl, 25 mM NaHCO_3_, pH 7.2) containing protease inhibitors (1 mM phenylmethylsulfonyl fluoride, 1 mM ethylenediaminetetraacetic acid, 0.2 mM Na-*p*-tosyl-L-lysine chloromethyl ketone and,1 mM leupeptine). The hemolymph was obtained from a small tear made laterally on the intersegmentary membrane of the two last abdominal segments. The first drop of perfused hemolymph was collected directly on a glass silanized tube (Thermo Scientific) placed on ice, and 100 µl of PBS containing the protease inhibitors were added to the tube. For each data point, at least four independent samples of hemolymph were collected from pools of five insects each.

### JH quantification

Certified standard solutions for JH III and its deuterated analog (JH III-D3) were obtained from Toronto Research Chemicals (Toronto, Canada). After hemolymph extraction, 10 µL of 6.25 ppb of JH III-D3 in acetonitrile were added to each sample, followed by 600 µL of hexane. Samples were vortexed for 1 minute, and spun for 5 minutes at 4 °C and 2000 g. The organic phase was transferred to a new silanized vial, dried under nitrogen flow, and stored at −20 °C. Dried extracts were re-suspended in 50 µl of acetonitrile, vortexed 1 minute, transferred to a new silanized vial with a fused 250 µL insert. JH III quantifications by high performance liquid chromatography coupled to electrospray tandem mass spectrometry (HPLC-MS/MS) were done as previously described by Ramirez *et al*.^14^. Briefly, we employed a HPLC-MS/MS workflow based on multiple reaction monitoring (MRM) using the two most abundant fragmentation transitions: 267-> 235 (primary) and 267-> 147 (secondary). In order to accurately quantify the amount of JH III present in the hemolymph, the heavy deuterated JH III analog (JH III-D3) was utilized as an internal standard to normalize recoveries during the sample preparation, extraction and analysis steps. An extraction recovery of near 55% was routinely observed regardless of the analyte concentration^14^.

### Statistical analysis

Statistical analyses were performed using the GraphPad Prism Software 3.03 (San Diego, CA, USA). The results are expressed as means ± SEM. Significant differences (*P* < 0.001) were determined with a one tailed students t-test performed in a pair wise manner or by one-way ANOVA followed by Tukey’s test.

## Supplementary information

Small and large An. albimanus mosquitoes sizes and follicles

## Data Availability

All data generated or analyzed during this study are included in this published article (and its supplementary information files).
