# Supplementary material for: Juvenile hormone controls ovarian development in female Anopheles albimanus mosquitoes
Source: Sci Rep. 2019 Feb 14;9:2127. doi: 10.1038/s41598-019-38631-6 (PMC6375968; doi:10.1038/s41598-019-38631-6)

Juvenile hormone controls ovarian development in female *Anopheles albimanus* mosquitoes

Salvador Hernández-Martínez, Víctor Cardoso-Jaime, Marcela Nouzova, Veronika Michalkova, Cesar E. Ramirez, Francisco Fernandez-Lima and Fernando G. Noriega.

Supplemental Fig S1.

Fig. S1. Small and large *An. albimanus mosquitoes*. A) Photography of a typical small (left) and (right) large female mosquito. Scale bar = 5 mm. **B**) Wing length of small (red) and large (black) *An. albimanus* females. I) laboratory females, II) Field-collected females. Each bar represents the mean ± SEM of wing length measurements from ten females. Asterisk denotes significant difference (unpaired t-test; *** P ≤ 0.001).

A

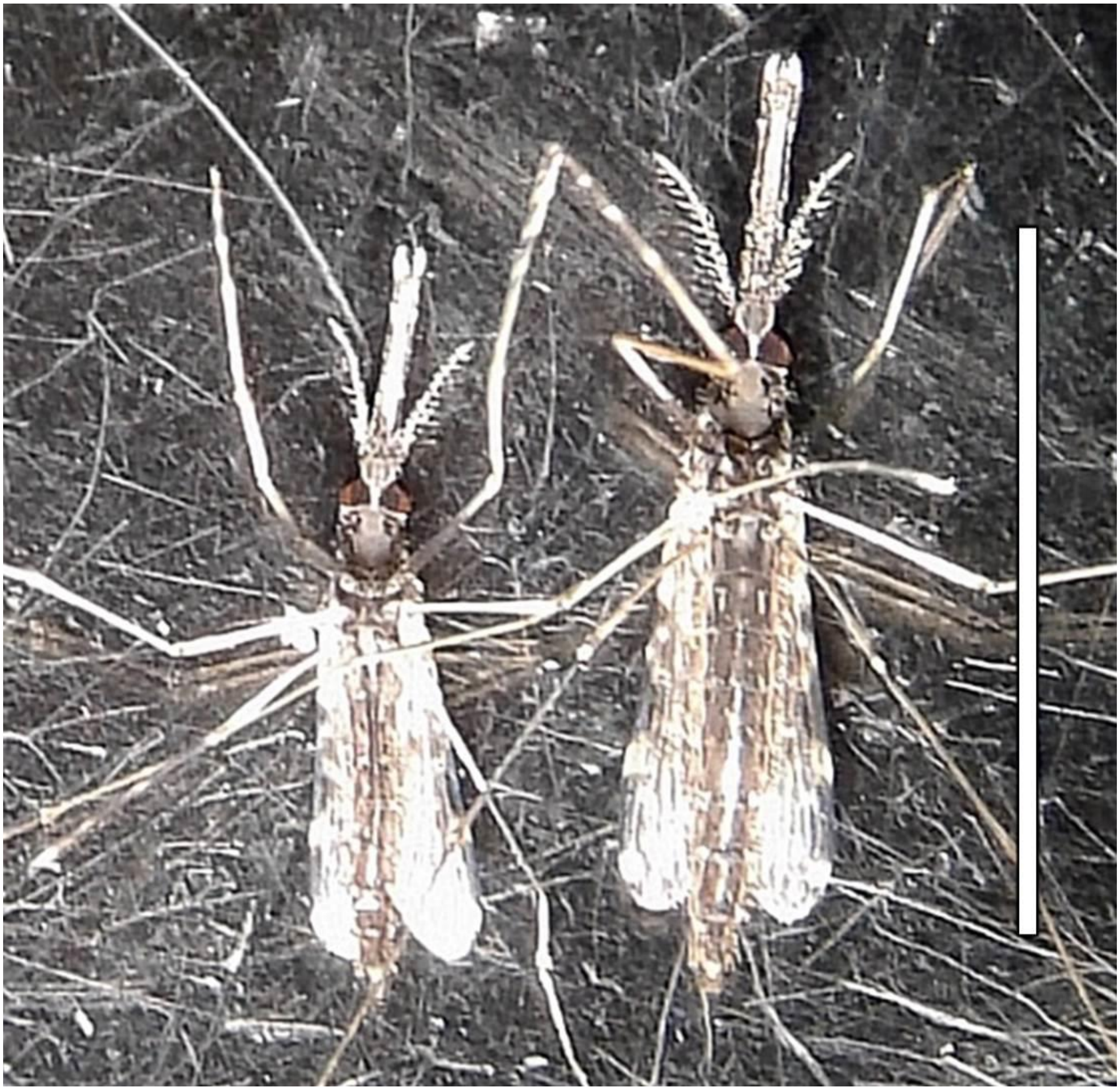

B

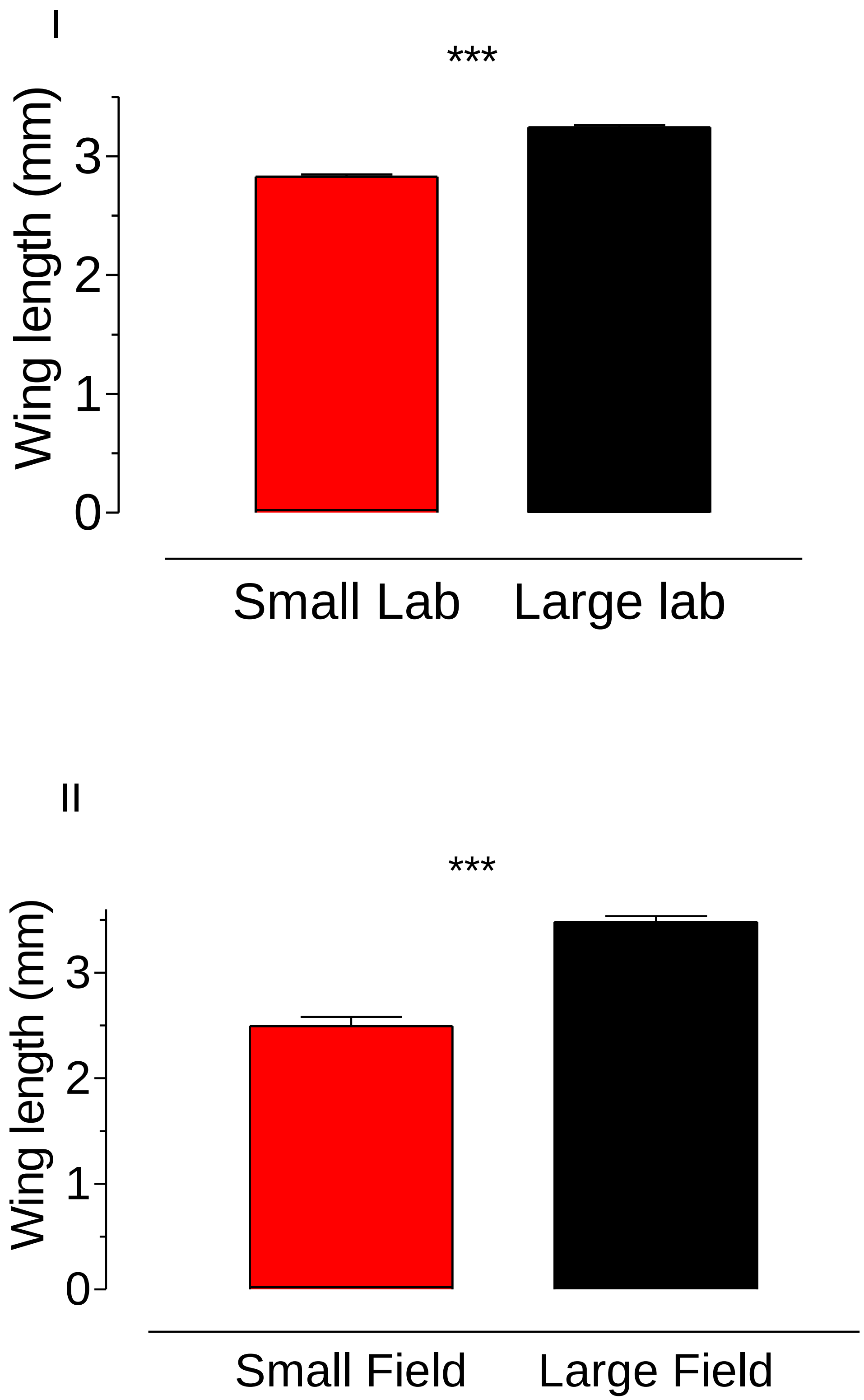
I
***
Wing length (mm)
0
1
2
3
Small Lab
Large lab
II
***
Wing length (mm)
0
1
2
3
Small Field
Large Field

Supplemental Fig S2.

Follicles in small and large *An. albimanus* mosquitoes. **A**) Follicles from small *An. albimanus* females at 24-36 h post-emergence. **B**) Follicles from large *An. albimanus* females at 24-36h post-emergence. We often observed the separation and development of secondary follicles to the ~40 mm size in sugar-fed large females by 24-36 h post-emergence, when the primary follicles have only reached their maximum "resting stage" size of 100 mm. Arrows, primary follicles; arrow-heads, secondary follicles. Scale bars = 50 μm.

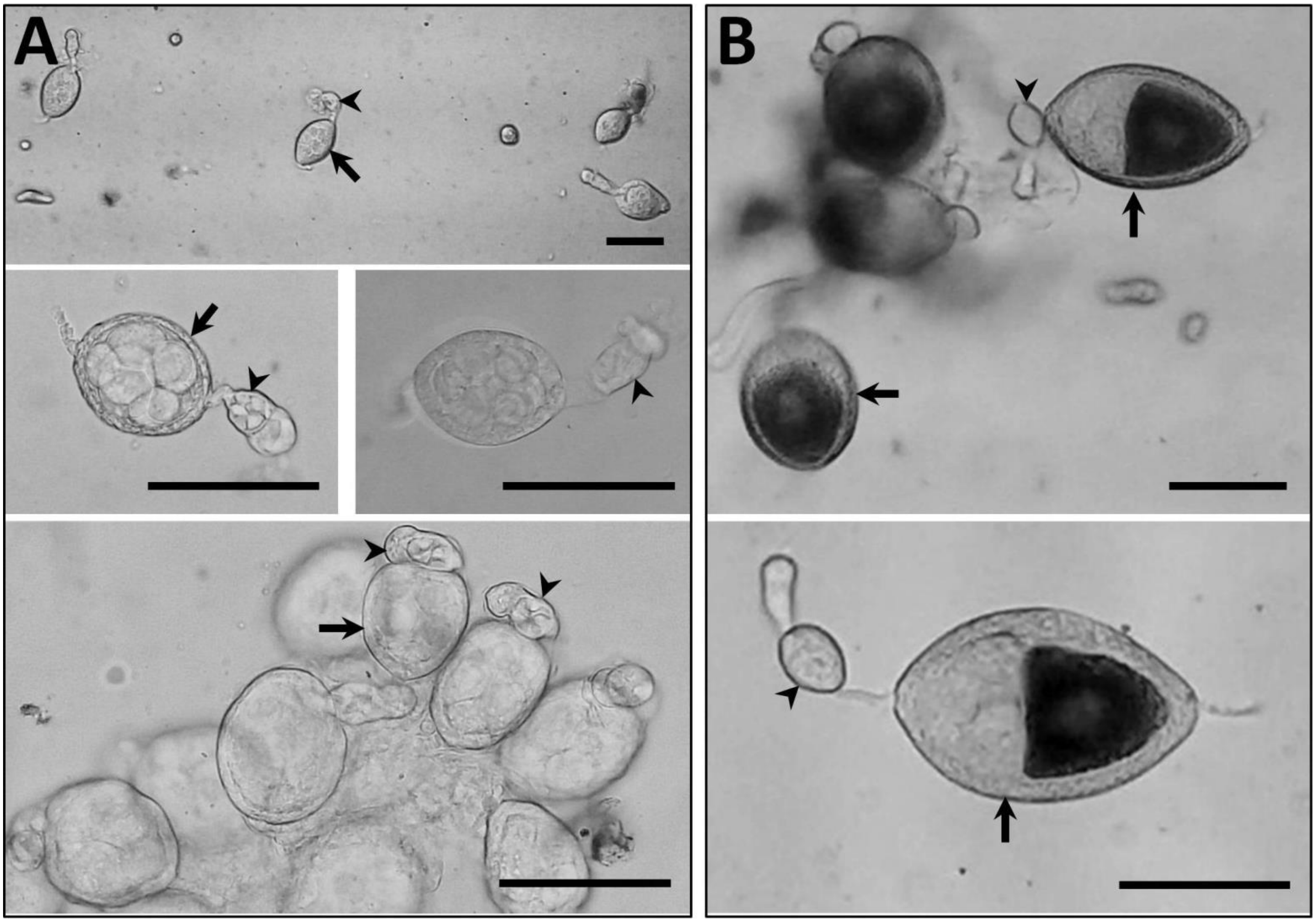

Supplement: Supplementary file 1 — Small and large An. albimanus mosquitoes sizes and follicles [file 41598_2019_38631_MOESM1_ESM.pdf]
